# The effect of a novel, digital physical activity and emotional well-being intervention on health-related quality of life in people with chronic kidney disease: trial design and baseline data from a multicentre prospective, wait-list randomised controlled trial (kidney BEAM)

**DOI:** 10.1186/s12882-023-03173-7

**Published:** 2023-05-02

**Authors:** C. G Walklin, Hannah M.L Young, E Asghari, S Bhandari, R. E Billany, N Bishop, K Bramham, J Briggs, J. O. Burton, J Campbell, E. M Castle, J Chilcot, N Cooper, V Deelchand, M. P.M Graham-Brown, A Hamilton, M Jesky, P. A Kalra, P Koufaki, K McCafferty, A. C Nixon, H Noble, Z. L. Saynor, C Sothinathan, M. W Taal, J Tollitt, D.C Wheeler, T. J Wilkinson, J.H Macdonald, S. A Greenwood

**Affiliations:** 1 Renal Therapies, King’s College Hospital NHS Trust, London, UK; 2 Leicester Diabetes Centre, Leicester General Hospital, Leicester, UK; 3 Department of Nephrology, Guy’s and St Thomas’ NHS Trust, London, UK; 4 Department of Nephrology, Hull University Teaching Hospitals NHS Trust, Hull, UK; 5 Department of Health Sciences, University of Leicester, Leicester, UK; 6 School of Sport, Exercise and Health Sciences, University of Loughborough, Loughborough, UK; 7 Department of Women’s Health, King’s College London, London, UK; 8 Faculty of Health, Education and Society, University of Northampton, Northampton, UK; 9 School of Physiotherapy, Department of Health Sciences, Brunel University, London, UK; 10 Institute of Psychiatry, Psychology & Neuroscience, King’s College London, London, UK; 11 Department of Nephrology, Royal Free Hospital, London, UK; 12 Department of Nephrology, Royal Exeter Hospital, Devon, UK; 13 Department of Nephrology, Nottingham NHS Trust, Nottingham, UK; 14 Department of Nephrology, Royal Hospital, Northern Care Alliance NHS Foundation Trust, Salford, UK; 15 Dietetics, Nutrition and Biological Sciences, Queen Margaret University, Edinburgh, UK; 16 Department of Nephrology, Barts Health NHS Trust, London, UK; 17 Department of Renal Medicine, Lancashire Teaching Hospitals NHS Foundation Trust, Preston, Lancashire UK; 18 Division of Cardiovascular Sciences, The University of Manchester, Manchester, UK; 19 School of Nursing and Midwifery, Queen’s University, Belfast, UK; 20 School of Sport, Health and Exercise Science, University of Portsmouth, Portsmouth, UK; 21 Department of Physiotherapy, Chelsea and Westminster NHS Trust, London, UK; 22 Centre for Kidney Research and Innovation, University of Nottingham, Nottingham, UK; 23 Department of Renal Medicine, University College London, London, UK; 24 National Institute of Health Research Leicester Biomedical Research Centre , Leicester, UK; 25 Institute for Applied Human Physiology, Bangor University, Bangor, Gwynedd UK; 26 Faculty of life sciences and medicine, King’s College London, London, UK

**Keywords:** KDQoL-SF, SF-36, e-health, Telemedicine, Digital health, Mixed-methods, Quality of life

## Abstract

**Background:**

Physical activity and emotional self-management has the potential to enhance health-related quality of life (HRQoL), but few people with chronic kidney disease (CKD) have access to resources and support. The Kidney BEAM trial aims to evaluate whether an evidence-based physical activity and emotional wellbeing self-management programme (Kidney BEAM) leads to improvements in HRQoL in people with CKD.

**Methods:**

This was a prospective, multicentre, randomised waitlist-controlled trial, with health economic analysis and nested qualitative studies. In total, three hundred and four adults with established CKD were recruited from 11 UK kidney units. Participants were randomly assigned to the intervention (Kidney BEAM) or a wait list control group (1:1). The primary outcome was the between-group difference in Kidney Disease Quality of Life (KDQoL) mental component summary score (MCS) at 12 weeks. Secondary outcomes included the KDQoL physical component summary score, kidney-specific scores, fatigue, life participation, depression and anxiety, physical function, clinical chemistry, healthcare utilisation and harms. All outcomes were measured at baseline and 12 weeks, with long-term HRQoL and adherence also collected at six months follow-up. A nested qualitative study explored experience and impact of using Kidney BEAM.

**Results:**

340 participants were randomised to Kidney BEAM (*n* = 173) and waiting list (*n* = 167) groups. There were 96 (55%) and 89 (53%) males in the intervention and waiting list groups respectively, and the mean (SD) age was 53 (14) years in both groups. Ethnicity, body mass, CKD stage, and history of diabetes and hypertension were comparable across groups. The mean (SD) of the MCS was similar in both groups, 44.7 (10.8) and 45.9 (10.6) in the intervention and waiting list groups respectively.

**Conclusion:**

Results from this trial will establish whether the Kidney BEAM self management programme is a cost-effective method of enhancing mental and physical wellbeing of people with CKD.

**Trial Registration:**

NCT04872933. Registered 5th May 2021.

**Supplementary Information:**

The online version contains supplementary material available at 10.1186/s12882-023-03173-7.

## Background

Chronic kidney disease (CKD) is a global health issue affecting ~ 700 million adults worldwide, conferring a substantial economic burden [1]. CKD is associated with elevated rates of cardiovascular and metabolic mortality risk [2], symptom burden [3] and sedentary behaviour [4, 5]. People with CKD also report physical inactivity [6], reduced physical function [7], and poor psychological wellbeing [8]. Together, these factors have a substantial impact on health-related quality of life (HRQoL) [9]. Indeed, people with CKD demonstrate significantly lower HRQoL when compared with people without CKD [5].

Reduced HRQoL and a reduced ability to participate in life events remains a primary concern for people living with CKD, irrespective of stage of disease, making it an important target for intervention [10–13]. Physical activity-focused rehabilitation has been identified within a recent umbrella review of systematic reviews as a promising intervention to increase HRQoL across the CKD trajectory [14]. In addition, it is also associated with improvements in cardiorespiratory fitness, muscle strength and body composition, muscular endurance, cardiovascular risk factors and dialysis-related symptoms [14]. Consequently, several recent national and international guidance documents conclude that PA recommendations for the general population be extended and encouraged across the CKD trajectory [15, 16]. Despite these recommendations, people with CKD do not routinely receive support for physical activity or emotional well-being as part of their routine clinical care and access to physical and psychological support services across the UK are highly variable [17, 18]. Barriers include a lack of appropriately qualified professionals, suitable facilities and difficulty with the wide geographical distribution of the CKD population who attend primary, secondary and tertiary care clinics, in addition to lack of funding [17].

Given that approximately 2.6 million people aged 16 years and older are living with CKD stage 3–5 in England, and that these numbers are predicted to rise to 4.2 million by 2036 [19, 20], increasing access to effective services to support physical and mental wellbeing and enhance HRQoL in this population has never been more critical [21]. This has only become more urgent as a result of the Coronavirus-19 (COVID-19) pandemic. CKD is a significant risk factor for more severe COVID-19 infection [22, 23], and many people with CKD were advised to shield at home. The longer-term ramifications of this have been an increase in sedentary behaviour and physical inactivity, leading to increased deconditioning and falls risk [24, 25], in addition to reduced HRQoL and increased anxiety and loneliness [24, 26].

Remote digital health interventions (DHIs) offer the potential to overcome many of these barriers and challenges, and there has been an increasing move towards models of digital healthcare for people with CKD [27]. Evidence for DHIs specifically in this population is, however, of low quality and is currently insufficient to guide practice [28]. There are also no studies assessing the clinical or cost-effectiveness of digital physical activity interventions [29]. Effective DHIs in other long-term multi-morbid conditions appear to be based upon an established theoretical model, including healthcare professional support and incorporating behaviour change techniques [29–31]. Kidney BEAM [32] is a theory-informed, evidenced-based online physical activity and emotional wellbeing self-management programme that was developed in response to the COVID-19 pandemic [33]. The programme is available across the UK, free at the point of contact and has been co-designed to allow people with CKD to learn about their condition and enhance their physical and mental wellbeing.

## OBJECTIVES

### Primary objective and outcome

To evaluate the effect of the Kidney BEAM DHI compared to a wait-list control group (usual care) on HRQoL, as assessed by the mental composite score (MCS) of the KDQoL SF1.3 questionnaire at 12 weeks [34].

### Secondary objectives and outcomes

The secondary objectives were to (i) establish whether Kidney BEAM could improve mental and physical wellbeing, physical function, fatigue and patient activation (a measure of an individual’s understanding, competence, and willingness to participate in care decisions and processes). (ii) Explore the perceptions and experiences of people who have used Kidney BEAM in the intervention group. Specifically, we proposed to examine whether Kidney BEAM delivered to adults with established CKD leads to improvements in the following outcomes:

- HRQoL – Physical- and kidney-specific HRQoL, as measured by the physical component and kidney-specific summary scores of the KDQoL SF1.3 questionnaire.
- Healthcare utilisation - Quality Adjusted Life Year (QALY) profiles for the cost-effectiveness analysis, generated using the EQ-5D-5L [3, 5].
- Fatigue - The Chalder Fatigue Questionnaire at 12 weeks [36].
- Life participation - The Work and Social Adjustment Scale (WSAS) [37, 38] .
- Patient activation- The PAM-13 [39–41].
- Self-reported physical activity - The Global Physical Activity Questionnaire (GPAQ) [42, 43].
- Depression and anxiety - The Patient-Health Questionnaire-4 (PHQ-4) [44].
- Field test of physical function - Sit-to-Stand in 60 seconds (STS60) [45–47].
- Biochemical measures - Blood markers of inflammation and immunity (C-Reactive Protein, Interleukin 6, haemoglobin), kidney function (creatinine, estimated glomerular filtration rate,) and HbA1c.

#### Sub-studies

i. An exploratory sub-study examining outcomes in participants with CKD who had COVID-19 or who have been left with CKD following acute kidney injury (AKI)-related to COVID-19 infection;
ii. A qualitative sub-study exploring digital inclusion and digital health literacy in participants with protected characteristics, or who may be more vulnerable to digital exclusion, and who have declined to participate in the study;
iii. A polycystic kidney disease (PKD) sub-study exploring outcomes and participant views of Kidney BEAM with additional PKD-specific education;
iv. A haemodialysis sub-study exploring the feasibility of different models of engagement with the Kidney BEAM programme specifically for participants recieving in-centre haemodialysis.
v. The Ex-Tab Sub-study which will explore the feasibility of engaging people who do not have access to a digital device or are not confident using the technology available to them, with a wifi-enabled digital device and additional training.

See Supplementary Material 1 for detailed information of the sub-studies.

## Methods

The reporting of the trial methods was informed by the *Standard Protocol Items: Recommendations for Interventional Trials (SPIRIT) Reporting Recommendations* [48], and other relevant reporting guidance [49–52]. See Supplementary Material 2 for World Health Organisation Trial dataset.

### Design, setting and participants

This trial is a prospective, investigator-led, multicentre randomised waitlist-controlled trial, with economic analysis and nested qualitative study.

**Fig. 1:**
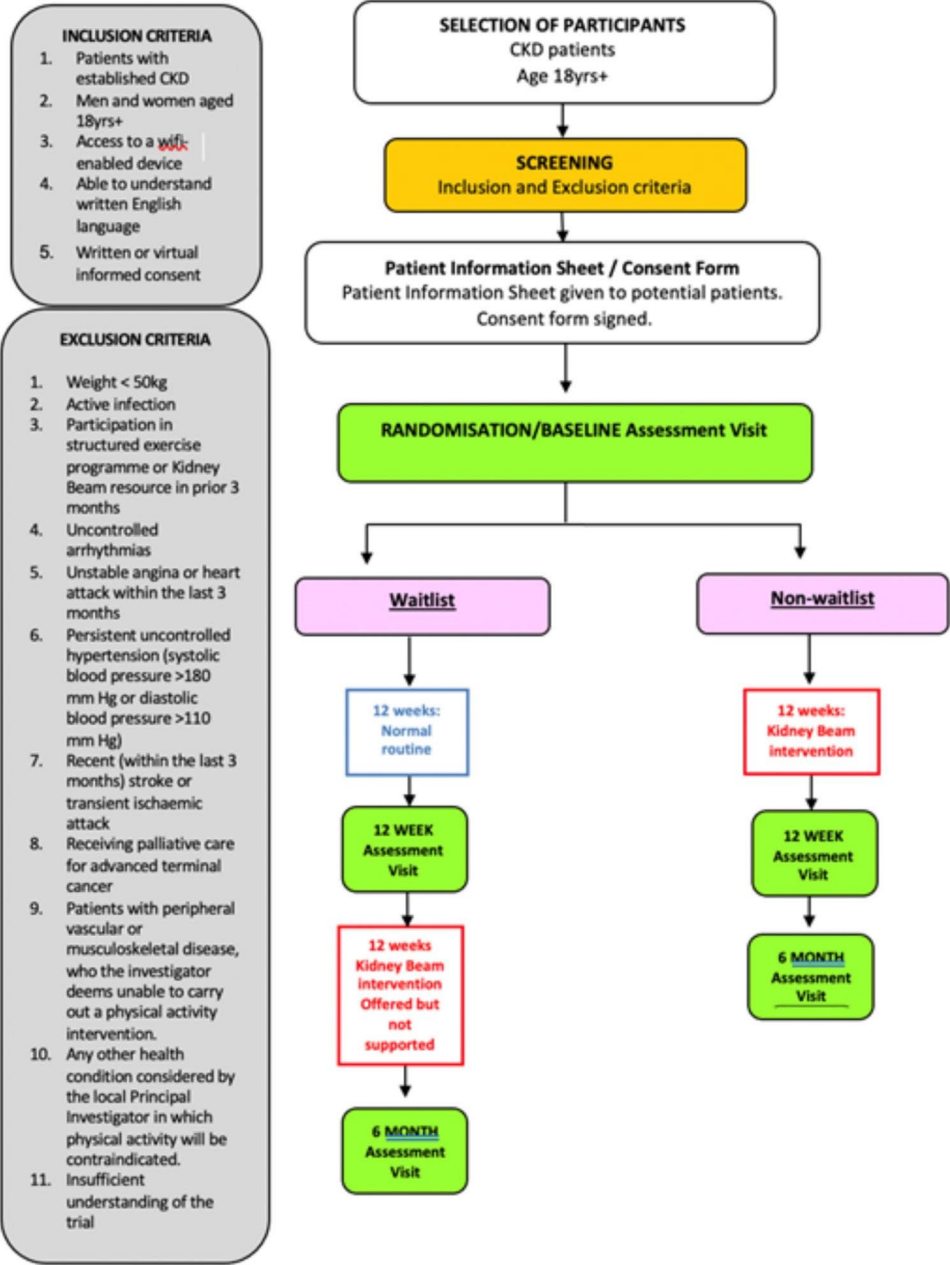
Trial flowchart

The trial design is summarised in Fig. 1. Ethics approval was granted by Bromley National Health System (NHS) Research Ethics Committee (REC) and the Health Research Authority (HRA) (21/LO/0243) and was prospectively registered (Trial Registration number NCT04872933) on 5th May 2021. The latest protocol (version 9.0) was dated 12/01/2023. All substantial and non-substantial amendments to the protocol were reviewed by the REC and HRA.

### Recruitment and eligibility

Recruitment took place at eleven National Health Service (NHS) hospitals within England from May 2021 to October 2022. A list of trial sites can be obtained from the clinical trials registry. Centres were selected to ensure the population was geographically, ethnically and socio-economically representative. Prospective participants were screened by their clinical team, and clinical records reviewed to confirm eligibility. Suitable adults were approached face-to-face during routine clinic visits, or via telephone, by trained research staff. Posters were also displayed at each kidney unit, allowing potential participants to contact the study team if they were interested.

Adults 18 years + of age with established CKD were eligible for inclusion in the trial. Participants who were unable to safely exercise, according to current American College of Sports Medicine guidelines [53], weighed < 50 kg, had previously participated in a structured exercise programme or Kidney BEAM within previous 3 months, or were unable to provide fully informed consent, were ineligible. The inclusion and exclusion criteria for the qualitative component mirrored those of the trial  (see Supplementary Material 3 for full inclusion/exclusion criteria). Only those in the intervention group were invited to participate in the interviews, as they had sufficient experience of using Kidney BEAM.

The trial was explained in full over the telephone or face-to-face, and a written information sheet subsequently provided via email. Following > 24 h, a member of the research team re-contacted the participant to answer any questions. No participation incentives were provided. Written informed consent was obtained via a secure online link (SurveyMonkey Ltd), or face-to-face at a routine clinic visit. Consent forms and Participants Information Sheets are included with Supplementary Material 4.

Following consent and baseline assessment, participants were randomised (1:1) to the intervention or wait-list control group using a validated web-based system (Sealed Envelope Ltd). Randomisation and treatment allocation was performed by an independent member of the research team, using an approach based on randomly permuted blocks. The allocation list was stored in a password-protected database.

Given the nature of the intervention, blinding of the healthcare professionals providing the programme and the participants was not possible. Outcome assessors were, however, blinded to treatment allocation. The statistical analysis plan was developed *a priori* by an independent statistician, unaware of treatment allocation, and approved by the Trial Steering Committee (TSC). Data entry and quality assurance will be undertaken by data entry clerks unaware of treatment allocation. Data cleaning and analysis of the primary outcome will be conducted by the independent statistician.

### Interventions

#### Kidney BEAM

The development and pilot testing of Kidney BEAM is described in detail elsewhere [54]. Briefly, Kidney BEAM is a digitally delivered physical activity and emotional well-being self-management intervention co-developed by King’s College Hospital NHS Foundation Trust (KCH) and the online platform team from BEAM, together with a diverse stakeholder group. The core development team consisted of a programme developer, two physiotherapists, a studio manager, an online marketing expert and a graphic designer. Content was developed by KCH with the study patient and public involvement group (PPI) and an extended team of physiotherapists and scientists with expertise in the design and delivery of kidney-specific exercise-based rehabilitation from the UK Kidney Research Consortium Exercise and Lifestyle Clinical Study group [55].

Kidney BEAM was rapidly co-designed in response to the COVID-19 pandemic. The intervention is guided by the Behaviour Change Wheel and the Behaviour Change Taxonomy [56, 57], and is adapted from the KCH-developed face-to-face renal rehabilitation 12-week programme [58]. and offers online live and on-demand exercise programmes, delivered by a range of credible healthcare professionals and patient champions, alongside email feedback and action planning, and online educational videos and blogs. The intervention did not undergo any major changes following the pilot period, but new content was added throughout the trial period, including new on-demand videos, blog entries and recorded live classes.

Kidney BEAM is currently free at the point of access in any setting or context, using a personal computer, tablet or smartphone. Participants were prompted to create a personal account through which they could book live classes, bookmark favourite components and track their online and offline physical activity. Participants could use Kidney BEAM *ad libitum*, but all were encouraged to attend at least twice weekly virtual renal rehabilitation sessions led by a renal physiotherapist and support worker in an online group setting (these staff were not involved in collecting outcome measures), or to complete the renal rehabilitation programme via a curated programme in the on-demand library, which followed the same programme of classes and education as those presented via the online live classes.

The ‘live’ sessions, and on-demand renal rehabilitation programme, were provided by the team at Kings College Hospital, London to all participants nationally as a rolling 12-week programme. Each session included a 10 min warm-up and cool-down consisting of general upper and lower limb mobility and stretching. Each session also provided 20–30 min of aerobic and resistance exercise training, which was delivered in standing and in a seated position so that people from different ability levels could participate. Modifications for fistula and fluid management were offered in each session. Hand and ankle weights, and the use of available home equipment such as filled water bottles and tins of food were suggested to progress resistance exercises. Additionally, 15 min of disease-specific education was offered weekly (see Supplementary Material 5).

Each participant was also encouraged to accumulate 150 min/week of moderate intensity aerobic activity or 75 min/week of vigorous activity, and to complete muscle resistance training on two days of the week [15]. A physiotherapy assistant, trained in motivational interviewing, encouraged participants on a weekly basis via telephone or email to achieve this target. The behaviour change techniques utilised, the time taken, and the staffing level of the research assistant were collected and will be reported alongside the results of the trial.

Participants were able to undertake these activities at any time that was convenient to them and their lifestyles, although ‘live’ classes were only available four times a week. Use of the DHI was monitored by the team at KCH, and participants prompted via phone or email on a weekly basis. These strategies were selected because they could easily be implemented within a real-world setting. Participants were also able to contact the team in the case of any technical or navigation issues using the Kidney BEAM website.

#### Adherence

Use (adherence) and/or non-use (attrition), were measured using a variety of metrics including number of classes attended, and time spent completing the classes. Functionality within Kidney BEAM allows for accurate measurement of physical activity minutes as it only records the time spent in the activity/session rather than total ‘time logged in’.

#### Waitlist control

Participants who were allocated to the wait-list control group did not participate in a structured exercise programme but were invited to use BEAM following the end of their involvement in the trial.

#### Usual care

Both arms continued with usual care in line with current guidance [59–61]. The majority of centres did not offer routine kidney-specific outpatient rehabilitation programmes. Participants were able to seek support with the management of any other related or unrelated medical conditions during the trial, and to be referred to additional services on an individualised and ad hoc basis. Health resource use was recorded and will be reported as a secondary outcome.

#### Internal pilot

The aim of the internal pilot was to establish the feasibility and acceptability of conducting an online RCT investigating the effects of Kidney BEAM. Feasibility data was integrated with interview data in a mixed-methods synthesis, which was assessed against a set of progression criteria, established *a priori.* The results were used to guide adaptation to the definitive trial design and to Kidney BEAM. The results of this pilot are reported in detail elsewhere [54].

### Assessment outcomes and their measurement

Baseline assessment was arranged at the participants’ convenience following written informed consent. Demographic and clinical characteristics including age; sex; ethnicity, cause of kidney disease and medications, were gathered, in addition to information relating to transplant or dialysis status and duration where applicable, from recent patient records. Recent information on height, weight, blood pressure, and heart rate were also gathered from routinely collected clinical information. Information on comorbidity, including diabetes status was also obtained and the Charlson Comorbidity Index (CCI) used to estimate the burden of comorbid disease [62]. The Clinical Frailty Scale (CFS), a validated risk stratification tool, identified vulnerable to severely frail participants [63].

All patient-reported outcome measures (PROMs) were captured directly from the participant, using a secure online questionnaire (SurveyMonkey Ltd) administered via email. Additionally, the KDQoL SF1.3, Eq. 5D-5 L, and healthcare utilisation were re-administered at six months follow-up and reported alongside Kidney BEAM metrics. This was to evaluate the longer term follow-up of participants following cessation of the intervention. As per SPIRIT recommendations [48], the schedule of enrolment, interventions, and assessments can be found in Table 1. Participants undertook the STS60 test at home with remote monitoring from an outcome assessor over the phone or via secure online video conferencing software. To promote retention participants were offered flexible appointments to fit around their work and life commitments. Reasons for non-retention (i.e., consent withdrawn; lost to follow-up) were documented.

**Table 1:** Schedule of Enrolment, Interventions, And Assessments

	Study period				
	Enrolment	Baseline and Allocation	Post-allocation		Close-out
TIMEPOINT	*-t*_*1*_	0	*t*_*1*_ _*(12 weeks)*_	*t*_*2*_ _*(six months)*_	*t*_*x*_
**ENROLMENT**:					
**Eligibility screen**	X				
**Informed consent**	X				
**Demographic data and medical history**	X				
**Allocation**		X			
**INTERVENTIONS**:					
***Kidney BEAM***					
***Usual care***					
**ASSESSMENTS**:					
**Height, weight, BMI, Clinical Frailty Scale**		X	X		
**Vital signs (Blood pressure & heart rate)		X	X		
**Full blood count		X	X		
**Creatinine, CRP, eGFR, Hb, IL-6, Albumin from routine bloods		X	X		
**Chalder Fatigue Questionnaire**		X	X		
**Work and Social Adjustment Scale (WSAS)**		X	X		
**KDQOL-36**		X	X	X	
**EQ-5D-5 L**		X	X	X	
**Sit to Stand 60 (STS60)**		X	X		
**Global Physical Activity Questionnaire (GPAQ)**		X	X		
**Physical Health Questionnaire (PHQ4)**		X	X		
**Patient Activation Measure (PAM-13)**		X	X		
**Qualitative interview**			X		
**Harms**					
**Kidney BEAM adherence and engagement metrics**			X	X	
**Healthcare utilisation questionnaire**		X	X	X	
***Post-COVID functional assessment tool questionnaire		X	X	X	
****Digital health literacy screening tool	X				
^*****^ **Hand grip Strength**		X	X		

Abbreviations: KDQoL: Kidney Disease Quality of Life, PHQ-4: Patient Health Questionnaire-4, WSAS: Work and Social Adjustment Scale, GPAQ: Global Physical Activity Questionnaire, PAM: Patient Activation Measure

** This procedure will only be a retrospective review of most recent available routine blood tests results and vital sign measurements. Results with date will be recorded by the study team. No new blood test will be collected from participants

*** Only participants who had history of COVID at time of consent will be assessed for Post-COVID functional assessment tool questionnaire at Baseline

****Only a sub set of patients from Kings College Hospital

^*****^Only in a subset of patients at Newcastle NHS Trust

### Follow-up visits

Participants completed follow-up measures at 12 weeks (± 2 weeks) and 6 months (± 2 weeks).

#### Health-related quality of Life Mental Component score (MCS) KDQOL

The primary endpoint for this study was the between-group difference in the mental composite score (MCS) of the KDQoL SF1.3 questionnaire at 12 weeks. The outcome was also completed at 6 months. The KDQoL SF1.3 instrument is a widely used, valid and reliable kidney disease-specific measure of HRQoL [64]. The KDQoL SF1.3 includes the 12-item short form survey which provides a summary score for both physical and mental HRQoL, and a kidney disease-specific score encompassing: burden, symptoms, problems and effects associated with kidney disease. These scores are totalled from 0 to 100, with higher score indicative of higher HRQoL.

#### Health-related quality of life

Physical and kidney specific HRQoL as measured by the physical component score (PCS) and kidney-specific summary scores of the KDQoL SF1.3, outlined above, were measured as secondary outcomes. Quality Adjusted Life Year (QALY) profiles for the cost-effectiveness analysis was generated using the EQ-5D-5 L at 12 weeks [35]. The EQ-5D-5 L determines health state descriptions across five components (mobility, self-care, usual activities, pain, anxiety and depression) combined with preference-weighted HRQoL index scores and has been widely used in the CKD population [65].

#### Healthcare utilisation

A healthcare usage questionnaire was developed to collect data on healthcare resource use and expenditure including visits to health professionals and inpatient hospital stays. Data relating to participant incurred costs associated with accessing Kidney BEAM, staff training and delivery was also collected, to assess the cost of the DHI. The questionnaire was completed by the trial research assistant during the online interview.

#### Fatigue

The severity of physical and mental fatigue was measured using the 11-item Chalder Fatigue Questionnaire at 12 weeks [36]. This questionnaire is a reliable measure of fatigue that has been across a range of long-term conditions, including CKD [66].

#### Life participation

The Work and Social Adjustment Scale (WSAS) is a valid and reliable self-report scale which was used to measure functional impairment in work, home management, social activities, private leisure activities and relationships [37, 38].

#### Patient activation

The PAM-13 is a widely-used, reliable and validated tool for measuring patient activation [39–41]. The PAM-13 consists of 13 items which assess a person’s knowledge, skills and confidence in managing their own health [39, 40].

#### Self-reported physical activity

The Global Physical Activity Questionnaire (GPAQ) is a valid and reliable measure of self-reported physical activity developed by the World Health Organisation (WHO) [42, 43]. The GPAQ assesses the intensity, duration and frequency of physical activity across three domains: occupational, transport-related and leisure-time physical activity. Data was cleaned, analysed and reported as per published WHO guidance.

#### Depression and anxiety

The Patient-Health Questionnaire-4 (PHQ-4) has good internal reliability and construct validity and was used as a composite measure of depression and anxiety symptoms [44].

#### Physical function

In addition to PROMs, participants undertook STS60, a field exercise test of physical function. The STS60 is a widely used and validated measure of muscle endurance [46, 47]. Participants were required to stand up and sit down from a chair as many times as possible for one minute. The height of the chair remained consistent between tests. The number of repetitions completed was recorded. Where the participant was unable to complete the test, the time at which they stopped was recorded, in addition to the number of repetitions completed. Participants unable to stand without the use of their arms or other support were recorded as unable to complete the test [45].

#### Biochemical measures

Blood markers of inflammation and immunity (CRP, IL-6), haemoglobin, kidney function (creatinine, estimated glomerular filtration rate) and HbA1c were obtained from recently routinely collected blood tests.

#### Harms

Serious adverse events were reported to the study sponsor. Any adverse events relating to Kidney BEAM were identified and documented at routine assessments, based on participant reports and primary or secondary care reports. In addition, episodes of care requiring hospitalisation were documented.

### Nested qualitative interviews

For the nested qualitative component, a purposive sample of participants who had been involved in the intervention arm were invited to participate in a one-to-one semi-structured interview. Semi-structured interviews were selected as they offered an open and flexible method for the in-depth exploration of participants’ individual experiences. Interviews occurred at two time points:

- At the end of the internal pilot phase, to further understand the acceptability of Kidney BEAM and to contextualise and expand upon the feasibility outcomes. The methods used, and results of these interviews are reported in detail elsewhere [54].
- On completion of the trial to explore participants’ experiences of using Kidney BEAM and its impact upon living with kidney disease, to further contextualise the results of the trial and to understand its potential longer-term use (‘final interviews’).

A topic guide for the final interviews (Supplementary Material 6) was developed in advance of the study by HMLY, EMC, RB and JB in partnership with the trial management group (TMG) and PPI representatives. The guide was informed by the RE-AIM framework, which is a comprehensive model assessing five dimensions important to intervention impact and sustainability [67, 68]. These are: reach (the number, proportion, and representativeness of those who use Kidney BEAM); effectiveness (the impact of the Kidney BEAM on key outcomes, and why these results are observed); adoption (the extent to which Kidney BEAM is accepted or initiated by target populations); implementation (how consistently Kidney BEAM is applied and whether it was delivered as intended) and maintenance (the extent to which Kidney BEAM is sustained and adopted into routine use) [67, 68]. The RE-AIM framework has been widely used and provides a robust means of enhancing the quality and impact of healthcare interventions, and accelerating their translation into practice [67, 68]. Given that BEAM is delivered remotely by a single team, adoption and implementation at the provider level is less relevant, therefore we focused upon the domains at the individual participant level, and explored the *potential* for longer-term maintenance following the end of the programme [69, 70]. The guide was piloted during the first three interviews. As only minor adaptations were made to question content and order, and these initial interviews were retained in the final analysis.

Interviews were conducted by experienced qualitative researchers (HMLY,EMC,RB,JB, CW) at an appointment separate from the other trial assessments, via telephone or secure online video conferencing software. Participants were interviewed as close to the completion of the full trial as possible, to enable them to comment fully on their experiences. Interviews were scheduled to take up to 60 min and were digitally audio-recorded.

### Sample size

#### For the primary outcome

A mean score of 50 arbitrary units (AUs) on the MCS is representative of the average score in the US general population [34]. Studies that have evaluated the MCS of the KDQoL SF1.3 HRQoL questionnaire in people living with kidney disease indicate that a clinically meaningful improvement in MCS is 3 AUs [34]. An estimated sample size of 106 participants in each group (total *n* = 212), based on an MCS with a mean (SD) of 45 ± 10 and correlation between repeated measures of 0.7, allows a clinically meaningful difference of 3AUs to be detected at 80% power and 5% alpha. In total, 304 participants were recruited to allow for potential attrition or non-compliance of 30%, which is reflective of other trials in this field [58, 71].

#### For the nested qualitative study

The sampling and sample size rationale for the internal pilot interviews is outlined elsewhere [54]. For the final interviews, a planned sample size of up to 30 participants was selected to ensure good representation of participants living with different stages of CKD and/or receiving different forms of kidney replacement therapy. Maximum variation sampling was initially used to ensure that participants are purposefully diverse [72], and important characteristics (e.g. age, gender, ethnicity) were monitored throughout recruitment and as analysis progressed to ensure that a representative and inclusive sample was recruited [73]. Data collection ceased at the point where information power was achieved [74].

### Statistical analysis plan

Statistical analyses will be undertaken by an independent statistician, according to a statistical analysis plan developed, and signed, prior to database lock. All analyses will use SPSS v28 or a later version if available.

#### Summary of baseline data and flow of participants

A CONSORT diagram showing the flow of participants through the study will be produced. Data will be checked for outliers and missing values and validated using the defined score ranges. Baseline characteristics of participants by group and overall will be provided: means and standard deviation/medians and interquartile range for numerical variables, depending on data distribution and numbers and percentages for categorical variables. Descriptive summaries will similarly be provided for each outcome variable, at 12 weeks and six months where applicable, by study group.

#### Analysis of the primary outcome

The primary objective will be analysed under *intention-to-treat* assumptions (i.e., regardless of whether or not they dropped out/were lost to follow-up or fully adhered to the programme (intervention group) for all those who had been randomly allocated to groups, using a Last Observation Carried Forward (LOCF) approach to missing data, which will produce a conservative assessment of effect. However, exploratory analyses for all outcomes will also be conducted using *per protocol* analyses where only those complying with the specified intervention protocol, and who have both baseline and 12-week outcome data will be included. This is to assess efficacy under ideal conditions.The primary outcome of the MCS of the KDQoL SF1.3 questionnaire at 12 weeks will be analysed using an ANCOVA model, using baseline MCS of the KDQoL SF1.3 questionnaire and age as covariates. If the assumptions for parametric testing are not met, the equivalent non-parametric equivalent tests will be performed.

#### Analysis of secondary outcomes

ANCOVA analysis will also be performed for the MCS KDQoL SF1.3 questionnaire at 6 months. Secondary endpoints at the 12-week and 6-month timepoint will also be analysed using an ANCOVA mixed model (with time and group interactions), with the baseline value of the variable and age, as covariates.

#### Handling of missing data

Questionnaire-based variables to be analysed are the KDQoL SF1.3, the EQ-5D-5 L, Chalder Fatigue Questionnaire, the WSAS, GPAQ, PAM-13 and the PHQ4. The number (%) with complete data will be reported. Where questionnaire-specific guidance is available for the treatment of missing items in the construction of scales, this will be used. Where such guidance is not available, the following guideline will be followed, i.e., scales will be pro-rated for an individual if 20% or fewer items are missing. For example, in a scale with ten items, prorating will be applied to individuals with one or two items missing. The average value for the eight or nine complete items will be calculated for that individual and used to replace the missing values. The scale score will be calculated based on the complete values and these replacements.

We expect baseline MCS KDQoL SF1.3 questionnaire data to be nearly complete, if not fully complete. It is unlikely that excluding any participants with missing baseline data will bias treatment effect estimates or lead to much loss of precision, if any, and so a complete case analysis will be undertaken in such a scenario [86]. If there is some missing data for the primary outcome of MCS KDQoL SF1.3 questionnaire at 12 weeks, several strategies will be considered. If missingness is below 5%, and missingness is not thought to be isolated to a particular clinically relevant subgroup, then a complete-case analysis (CCA) will be performed [75]. If there is more than 5% missing data, then alternative approaches will be used. If participants with missing 12-week MCS KDQoL SF1.3 questionnaire data have post-randomisation variables predictive of missingness of 12-week MCS (e.g., six-month MCS) *and* predictive of the 12-week MCS values themselves, then a linear-mixed model (LMM) will be performed. This will assume that the missing data is missing at random (MAR) conditional on these post-randomisation variables. Unlike CCA, a LMM can include potentially informative post-randomisation variables without impacting the treatment effect estimates. LMMs also perform similarly to multiple imputation (MI) in such a multivariate scenario [75], whilst not requiring completeness of the post-randomisation auxiliary variables. Although it is unlikely that most participants with missing 12-week MCS will have six-month MCS data, there may be other post-randomisation variables associated with 12-week MCS and its missingness. If such post-randomisation variables are not present, patterns of missingness in *baseline* variables will be assessed. If the data is thought to be MAR conditional on observed baseline variables, then a complete-case analysis (CCA) adjusted for variables predictive of missingness will be performed. Under the MAR assumption, this gives unbiased and efficient treatment effect estimates, and generally performs better than alternative methods like MI.

#### Exploratory analyses

Exploratory analyses will include regression analyses between secondary outcome measures.

The effect of possible moderating variables will be investigated, e.g.:

- Moderation analyses using Clinical Frailty Scale as moderator.
- Moderation analyses using Chalder Fatigue Questionnaire as a moderator.

The effect of mediationg variables will be investigated, e.g.:

- Whether the change in the primary outcome (MCS) or the change in PCS is mediated by the change in functional capacity (STS60).
- Whether the change in the primary outcome (MCS) is mediated by the change in patient activation (PAM-13).

#### Substudy analyses

The same statistical analyses as described above will be applied for the sub-group datasets.

#### Interim analyses

An interim database lock and analysis will be completed after all data have been entered for the Baseline and 12-week visit (Primary Outcome timepoint). Analysis of the primary outcome and other selected 12-week outcomes will be conducted using the same approaches as described above for the main end-of-study analyses. An unblinded analysis of the 6-month outcomes and any remaining 12-week outcomes will be conducted at the study end, after the final database lock. The purpose of an interim analysis would be to allow early dissemination of the results for the study’s primary outcome. It is not intended to modify the study following this analysis, and no further analysis of the primary outcome or selected 12-week outcomes is planned. Therefore, the significance level for this interim analysis will not be adjusted to allow for multiple testing.

#### Analysis of interview data

The recorded interviews were professionally transcribed verbatim. Analysis of the pilot interviews is described elsewhere [54]. Analysis of the main interviews occurred in parallel to quantitative data collection, and was informed methodologically by the Framework approach [76] and theoretically by the RE-AIM framework [67]. The framework approach was primarily selected to support the management of a large dataset, overseen by multiple researchers.

Briefly, researchers with qualitative research experience will independently familiarise themselves with the interview data by listening to the audio-recordings and reading and re-reading the transcribed data. Following this, the qualitative research team will independently code the first three interviews. A combined deductive and inductive approach to coding will be taken. This will allow themes and codes to be pre-determined based on the domains of the RE-AIM framework, which aligns directly to the aims of the trial, but also ensures unexpected codes and themes can be constructed directly from the participants’ accounts and experiences. The team will meet to discuss and refine these initial codes, culminating in an agreed working analytical framework that will then be systematically applied to the remaining transcripts. Throughout this stage the research team will meet regularly to review and refine the framework as indicated. NVivo (QSR International) software will be used to manage the qualitative data and facilitate these initial stages of analysis. Following the application of the analytic framework, data will be charted into a matrix, which will summarise the data from each participant by theme. This stage will be managed using Excel software (Microsoft Ltd). The developed matrices will be discussed with the qualitative research team, before being used to interpret the data by comparing data both across and within cases.

All members of the qualitative research team will keep a reflexive diary in which they will reflect upon how their backgrounds, experiences, and knowledge may influence the qualitative research process and the interpretation of the data [76, 77]. They will also document initial interpretations and links between codes and themes [76]. These records will help to create an ‘audit trail’ of analytical decisions and the development of codes and themes [77].

#### Mixed-methods analysis

Following separate qualitative and quantitative data analyses the results will be integrated a ‘joint display’ [78]. Using RE-AIM as a sensitising framework, this joint display will combine both data sets in a tabulated form to comprehensively assess the ways in which the qualitative and quantitative data sets agreed (confirmed), complemented (offered an expanded explanation) or contradicted each other [78, 79]. This mixed methods approach will provide a more nuanced understanding of how and why the intervention lead to the results observed [78].

### Health economic analyses

#### Objective(s) of economic evaluation

The primary objective of the health economic evaluation is to estimate the cost-effectiveness of Kidney BEAM versus current therapy for people with CKD. Within-trial evaluations will assess cost-effectiveness of Kidney BEAM at 12 weeks and six months. After six months participants will have unsupported access to Kidney BEAM.

#### Overview of economic analysis

The within-trial economic analyses will be performed using individual patient level data collected from the trial. The analytical approaches will take the form of cost-effectiveness and cost-utility analysis. Based on trial evidence, incremental cost-effectiveness (and cost-utility) ratios will be calculated by taking a ratio of the difference in the mean costs and mean effects (or utility measure). The trial will be conducted in the UK, which has an NHS providing publicly funded healthcare, primarily free of charge at the point of use. The primary economic analysis will be from the NHS and personal social services (PSS) perspective. The primary economic analysis will compare the costs and consequences of each arm over the first 12 weeks after randomisation. A secondary analysis will extend this to compare costs and benefits over six-month follow-up period from randomisation. Stata version 17 or higher will be used for exploratory analysis and Stata and/or WinBUGS for the main statistical analysis.

### Data monitoring and quality assurance

The trial was coordinated by the TMG. A TSC was also established to oversee conduct and progress of the trial. The TSC met via teleconference prior to commencement of the study, after ethics approval, and every six months thereafter to assess study conduct and recruitment. Formal data monitoring was performed by a Data Safety Monitoring Committee (DSMC). The members of the DSMC were independent from the sponsor and investigators. The DSMC met via teleconference every four months to review accumulating data relating to harms and the primary outcome and advise the TSC whether the trial should be modified or discontinued.

### Patient and public involvement

The PPI group for this study comprised six people with lived experience of CKD (*n* = 3, 50% male; 53 ± 17 years; *n* = 3,50% White British; *n* = 2; 33% pre-dialysis CKD, *n* = 2, 33% dialysis, *n* = 2, 33% transplanted).The PPI group was involved in co-developing Kidney BEAM, but also early in the ethical approval stages. They contributed to the writing of lay summaries, providing patient perspectives on data collection procedures, ethical issues, and trial dissemination plans. They assisted in the preparation of trial documentation and interview topic guides. During the study, members of the PPI group attended regular PPI panel meetings.

## Results

### Baseline characteristics of randomised participants

The baseline charcteristics are presented in Tables 2 and 3. Three hundred and forty participants were randomised to Kidney BEAM (*n* = 173) and waiting list (*n* = 167) groups. Groups were well-matched for age, gender, ethnicity and BMI. The stage of chronic kidney disease for both the Kidney BEAM and waiting list groups were comparable, although stage 3B was higher in the non waiting list group (26.2%) than the waiting list group (18.6%) and stage 5 was lower in the Kidney BEAM group (21.5%) than the waiting list group (25.1%). There was a higher proportion of current smokers in the waiting list group (6.6%) compared to the intervention group (2.9%).

**Table 2:** Baseline demographic data

		All N = 340		Non Waiting list N = 173		Waiting list N = 167
	**n**		**n**		**n**	
Age (years)*	340	53.8 (13.5)	173	53.9 (13.6)	167	53.8 (13.5)
**Sex**	340		173		167	
Male		185 (54%)		96 (55%)		89 (53%)
Female		155 (46%)		77 (45%)		78 (47%)
**Ethnicity**	339		173		166	
Black		39 (11.5%)		20 (11.6%)		19 (11.4%)
White		254 (74.9%)		127 (73.4%)		127 (76.5%)
Asian		39 (11.5)		22 (12.7%)		17 (10.2%)
Biracial		7 (2.1%)		4 (2.3%)		3 (2.1%)
**BMI (kg/m** ^**2**^ )*	327	28.4 (24.8, 33.3)	165	27.9 (24.7, 33.4)	162	28.8 (24.9, 33.0)
**Smoking**	339		172		167	
Current		16 (4.7%)		5 (2.9%)		11 (6.6%)
Former		130 (38.3%)		77 (44.8%)		53 (31.7%)
Never		193 (56.9%)		90 (52.3%)		103 (61.7%)
**Alcohol consumption**	339		172		167	
More than recommended		26 (7.7%)		14 (8.1%)		12 (7.2%)
Less than recommended		174 (51.3%)		89 (51.7%)		85 (50.9%)
Non-drinker		139 (41%)		69 (40.1%)		70 (41.9%)
SBP (mmHg)*	307	136.5 (18.4)	154	135.3 (19.3)	153	137.8 (17.5)
DBP (mmHg)*	307	79.7 (10.7)	154	78.6 (11.1)	153	80.7 (10.2)
Heart rate (bpm)*	207	77.6 (14.7)	103	77.8 (14.6)	104	77.3 (14.8)
**Medical History**	340		173		167	
CVA		8 (2.4%)		8 (4.6%)		4 (2.4%)
MI		8 (2.4%)		3 (1.7%)		5 (3%)
Diabetes		76 (22.4%)		37 (21.4%)		39 (23.4%)
Hypertension		235 (69.1%)		115 (68.9%)		120 (69.4%)
**Cause of kidney disease**	340		173		167	
Diabetic nephropathy		31 (9.1%)		13 (7.5%)		18 (10.8%)
Hypertension		38 (11.2%)		21 (12.1%)		17 (10.2%)
Nephrosclerosis		1 (0.3%)		1 (0.6%)		0 (0%)
IgA nephropathy		39 (11.5%)		18 (10.4%)		21 (12.6%)
Tubulointerstitial nephritis		5 (1.5%)		2 (1.2%)		3 (1.8%)
PKD		60 (17.6%)		31 (17.9%)		29 (17.4%)
Obstructive nephropathy		7 (2.1%)		2 (1.2%)		5 (3%)
Medullary sponge kidney disease		0 (0%)		0 (0%)		0 (0%)
Membranous nephropathy		5 (1.5%)		5 (2.9%)		0 (0%)
Lupus nephritis		5 (1.5%)		4 (2.3%)		1 (0.6%)
Unknown		65 (19.1%)		33 (19.1%)		32 (19.2%)
Other		84 (24.7%)		43 (24.9%)		41 (24.6%)
**CKD stage**	339		172		167	
Stage 2		55 (16.2%)		27 (15.7%)		28 (16.8%)
Stage 3 A		62 (18.3%)		29 (16.9%)		33 (19.8%)
Stage 3B		76 (22.4%)		45 (26.2%)		31 (18.6%)
Stage 4		67 (19.8%)		34 (19.8%)		33 (19.8%)
Stage 5		79 (23.3%)		37 (21.5%)		42 (25.1%)
Transplant vintage (months)**	340	0 (0, 34.75)	173	0 (0, 37.5)	167	0 (0, 32)
Dialysis vintage (months)**	339	0 (0, 2)	173	0 (0, 4.5)	166	0 (0, 0)
HBA1C (mmol/mol)**	124	39 (35, 48)	64	38.5 (34.25, 49.75)	60	39 (36, 47)
Creatine (mmol/L)**	332	159 (106, 292.75)	170	159 (108.75, 279.25)	162	160.5 (106, 330)
CRP (mg/L)**	169	4 (2, 9)	92	3.9 (2, 9.7)	77	4 (2, 9)
Hb (g/L)**	326	123 (111, 137)	165	125 (113, 137)	161	119 (109, 136.5)
**eGFR (mL/min/1.73m** ^**2**^ )**	312	36 (18, 53)	162	36 (19, 55.25)	150	36 (16.75, 52)
**Clinical Frailty Scale**	340		173		167	
Very fit		38 (11.2%)		16 (9.2%)		22 (13.2%)
Well		109 (32.1%)		55 (31.8%)		54 (32.3%)
Managing well		149 (43.8%)		77 (44.5%)		72 (43.1%)
Vulnerable		37 (10.9%)		21 (12.1%)		16 (9.6%)
Mildly frail		5 (1.5%)		3 (1.7%)		2 (1.2%)
Moderately frail		2 (0.6%)		1 (0.6%)		1 (0.6%)
Severely frail		0 (0%)		0 (0%)		0 (0%)
Very severely frail		0 (0%)		0 (0%)		0 (0%)
Terminally ill		0 (0%)		0 (0%)		0 (0%)

Total number of available data (n), *mean and standard deviation, **median and interquartile ranges, number (n) or percentage (%) for all participants and for the two randomised groups

Abbreviations: BMI: body mass index, SBP: systolic blood pressure, DBP: diastolic blood pressure, CVA: cerebrovascular accident, MI: myocardial infarction, PKD: polycystic kidney disease, HBA1C: glycated haemoglobin, CRP: C-reactive Protein, Hb: haemoglobin, eGFR: estimated glomerular filtration rate, CFS: Clinical Frailty Scale

**Table 3:** Baseline outcome measure data

		All N = 340		Non waiting list N = 173		Waiting list N = 167
	n		n		n	
Sit to stand 60 (number)**	340	23 (18, 28)	173	23 (16.5, 27)	167	23 (18, 28)
KDQoL**	340		173		167	
Symptom/ problem list	283	81.2 (72.7, 93.2)	140	79.5 (68.7, 90.9)	143	81.8 (72.7, 93.2)
Effects of kidney disease	327	81.2 (56.2, 93.7)	166	75.0 (50.0, 90.6)	161	81.2 (62.5, 93.7)
Burden of kidney disease	339	68.7 (31.2, 87.5)	172	56.2 (31.2,81.2)	167	75.0 (37.5, 87.5)
Work status	338	50 (0, 100)	171	50 (0, 100)	167	100 (50, 100)
Cognitive function	339	80 (66.7, 93.3)	172	76.7 (60.0, 86.7)	167	86.7 (66.7, 93.3)
Quality of social interaction	339	73.3 (60.0, 86.7)	172	73.3 (60.0, 86.7)	167	73.3 (66.7, 86.7)
Sexual function	204	37.5 (0, 100)	102	31.2 (0, 100)	102	50 (0, 100)
Sleep	337	57.5 (42.5, 71.25)	171	57.5 (42.5, 71.25)	166	58.7 (42.5, 72.5)
Social support	308	83.3 (66.7, 100)	158	83.3 (66.7, 100)	150	83.3 (66.7, 100)
Dialysis staff encouragement	147	87.5 (50, 100)	78	87.5 (50, 100)	69	87.5 (50, 100)
Overall health	338	60 (50, 80)	171	60 (50, 80)	167	60 (50, 80)
Patient satisfaction	181	83.3 (50, 100)	93	83.3(66.7, 83.3)	88	83.3 (50, 100)
Physical functioning	337	70 (35, 90)	171	65 (30, 90)	166	72.5 (43.7, 90.0)
Role - physical	337	50 (0, 100)	171	50 (0, 100)	166	50 (0, 100)
Pain	338	67.5 (45.0, 90.0)	172	66.2 (45.0, 80.0)	166	68.7 (45.0, 90.0)
General health	337	40 (25, 55)	171	35 (25, 55)	166	40 (25, 56.2)
Emotional wellbeing	337	72 (56, 84)	171	68 (52, 84)	166	72 (56, 88)
Role - emotional	337	66.7 (16.7, 100)	171	66.7 (0, 100)	166	33.3(100,100)
Social function	338	62.5 (37.5, 87.5)	172	62.5 (37.5, 87.5)	166	62.5 (37.5, 90.6)
Energy/fatigue	337	45 (25, 60)	171	40 (25, 55)	166	45 (25, 65)
SF-12 Physical Composite	337	40.1 (31.5, 51.3)	171	39.9 (29.6, 50.0)	166	40.9 (32.4, 52.2)
SF-12 Mental Composite	337	47.3 (37.0, 54.4)	171	45.0 (37.0, 54.0)	166	48.0 (37.0, 55.0)
Chalder Fatigue Scale**	339	15 (11, 20)	172	16.5 (11.0, 20.75)	167	14.0 (11.0, 18.0)
PHQ-4**	339	2 (0, 4)	172	2 (0, 5)	167	2 (0, 4)
WSAS Total**	321	9 (1.5, 18)	161	10 (3.5, 20)	160	8 (0, 16)
GPAQ Total physical activity MET.mins/wk**	231	1440 (600,3840)	111	1440 (480, 3840)	120	1440 (720, 3870)
**MET physical activity guidelines (> 600MET.min/wk)**	231	171 (74%)	111	79 (71.2%)	120	92 (76.7%)
Total physical activity mins/day**	231	51.4 (21.4, 137.1)	111	51.4 (17.1, 137.1)	120	51.4 (25.7, 138.2)
Work physical activity mins/day**	81	85.7 (25.7, 240)	34	173.6 (31.1, 342.9)	47	68.6 (21.4, 176.0)
Travel physical activity mins/day**	133	25.7 (12.9, 51.4)	56	27.1 (13.9, 60.0)	77	25.7 (12.9, 51.4)
Recreation physical activity mins/day**	149	30.0 (17.1, 60.0)	72	27.1 (13.2, 51.4)	77	32.1 (17.1, 68.6)
Sedentary mins/day**	307	420 (300, 600)	152	420 (300, 600)	155	420 (300, 600)
PAM level*	340		173		167	
Disengaged and overwhelmed		48 (14.1)		30 (17.3)		18 (10.8)
Becoming aware but still struggling		66 (19.4)		30 (17.3)		36 (21.6)
Taking action and gaining control		108 (31.8)		58 (33.5)		50 (29.9)
Maintaining behaviours and pushing further		118 (34.7)		55 (31.8)		63 (37.7)
PAM Scale**	340	63.1 (51.0, 77.7)	173	60.6 (51.0, 75.0)	167	65.5 (51.0, 77.7)

Total number of available data (n), *mean and standard deviation, **median and interquartile ranges [Q1,Q3], number (n) or percentage (%) for all participants and for the two randomized groups. Abbreviations: KDQoL: Kidney Disease Quality of Life, PHQ-4: Patient Health Questionnaire-4, WSAS: Work and Social Adjustment Scale, GPAQ: Global Physical Activity Questionnaire, MET: Metabolic Equivalent of Task, PAM: Patient Activation Measure

The KDQoL SF1.3 MCS and PCS were similar in both groups. Likewise, the STS60 and the PAM scale were well matched.

### Prior cardiovascular events and risk factors

A history of major adverse cardiovascular events affected the minority of participants. History of diabetes and hypertension were similar between both randomised groups. Prior stroke was higher in the Kidney BEAM group with *n* = 8 (4.6%) participants compared with *n* = 4 (2.4%) participants in the waiting list group.

## Discussion

Adequately powered studies that investigate the clinical value and economic viability of an online physical and emotional wellbeing resource for the improvement of HRQoL in people living with CKD are needed [28]. Investigating the effects of a DHI, such as Kidney BEAM, will provide insight into a potential model to deliver routine physical and emotional wellbeing to a population who are not routinely offered this valuable type of care and align to the NHS long-term plan [80].

The baseline demographic data of the Kidney BEAM trial cohort revealed a representative sample from all ethnic origins. There is a known under-representation of ethnic minorities in clinical trials [81–83], and it is reassuring that our sample is representative of the clinical population [20]. All baseline clinical values, including the KDQoL SF1.3 scores, STS60 test scores and PAM-13 were within normal parameters and were similar to values in previous exercise studies [71, 84]. Importantly, baseline values for the primary outcome and key secondary outcomes were similar across both randomised groups, as was the stage of CKD and age of randomised participants in the trial.

The results of the Kidney BEAM trial will address a significant knowledge gap in the promotion and use of DHIs that promote self-management of physical and emotional health for people with CKD, and will establish whether Kidney BEAM offers a cost-effective means of enhancing mental and physical wellbeing of people with CKD. Kidney BEAM represents a potential means of delivering physical activity based self-management to people living with CKD across a wide geographical area, addressing a substantial gap in the existing clinical care of this population.

## Electronic supplementary material

Below is the link to the electronic supplementary material.

Supplementary Material 1

Supplementary Material 2

Supplementary Material 3

Supplementary Material 4

Supplementary Material 5

Supplementary Material 6

## Data Availability

Findings from the study will be disseminated at national and international conferences. All baseline data generated or analysed during this study are included in this published article.
