# Supplementary material for: The effect of a novel, digital physical activity and emotional well-being intervention on health-related quality of life in people with chronic kidney disease: trial design and baseline data from a multicentre prospective, wait-list randomised controlled trial (kidney BEAM)
Source: BMC Nephrol. 2023 May 2;24:122. doi: 10.1186/s12882-023-03173-7 (PMC10152439; doi:10.1186/s12882-023-03173-7)
Supplement: Supplementary file 1 — Supplementary Material 1 [file 12882_2023_3173_MOESM1_ESM.docx]

**Supplementary material 1: Kidney Beam Trial Sub-studies:**

### COVID-19 Sub-study

Outcomes for people with advanced Chronic Kidney Disease (CKD), transplants and on dialysis following COVID-19 infection are amongst the worst of all long-term conditions^1 2^.Not only are mortality rates significantly higher than in the general population, but people with CKD are more likely to develop the most severe complications of COVID-19 including physical deconditioning from acute illness^3^. The long-term effects of COVID-19 on physical activity, cardio-respiratory fitness and sarcopaenia are unknown for patients with CKD. Additionally, acute kidney injury (AKI) complicates the recovery of up to 25% of people admitted to intensive care units with COVID-19 and a proportion of patients that survive will have varying degrees of CKD^4^. Programs of post-COVID rehabilitation are only just beginning to emerge^5^ and this exploratory sub-study will inform the design of more bespoke studies and interventions to meet the needs of post-COVID CKD patients and those recovering from the effects of post-COVID AKI.

Participants who have had COVID-19, or have been left with CKD following AKI-related to COVID-19 infection, will be included. This will be established at baseline using review of medical records, or by asking patients whether they have had a positive test for COVID-19.

For those who confirm they have had a positive test an assessment of the severity of their COVID will be made in the following way:

- COVID positive, managed at home not requiring hospital admission
- COVID positive, required hospital admission but no oxygen therapy
- COVID positive, required hospital admission and oxygen therapy
- COVID positive, required hospital admission and non-invasive ventilation
- COVID positive, required hospital admission and admission to intensive care

Additional details of the admission, including length of stay and time since discharge will be recorded. Subjects will continue in the study in the same way but will complete the post-COVID functional status tool questionnaire at baseline and follow-up in addition to other outcome measures^6^.

### Digital inclusion qualitative sub-study

Digital health interventions (DHIs) such as Kidney BEAM have the potential to provide cost-effective, and sustainable self-management support to a demographically and geographically diverse population of people^7^. For people with CKD, who live with a high burden of healthcare utilisation, DHIs also have the potential to reduce the burden associated with living with a long-term conditions^8^.

Existing reviews across long-term conditions indicate that although they may lead to improvement in clinical outcomes, they are most often utilised by people who are digitally and health literate with access to technology^8^ ^9^. Despite this, data suggests that 20% of the general population do not possess fundamental digital skills, whilst others do not have access to the appropriate equipment and infrastructure, or the motivation and confidence to use DHIs^7 9 10^. They may perceive DHIs as *more* burdensome if they are challenging to use^7 9 10^. Digital exclusion is closely associated with health inequality, and often those who stand to benefit the most from such interventions are unable to access them^10^. Given the COVID-19 pandemic has accelerated the development and introduction of DHIs into routine practice, consideration of the impact of digital exclusion for particular groups of people is warranted^8^. To date there has been little exploration of digital exclusion in relation to online physical activity and emotional wellbeing self-management programmes, and the specific barriers that exist for people with CKD when accessing these types of interventions. Not all patient populations are affected in the same way, or experience the same barriers, making understanding the perspectives of those who do not participate in the trial for reasons relating to digital exclusion particularly important when evaluating Kidney BEAM^10 11^.The aim of this qualitative sub-study is to explore challenges relating to digital inclusion and digital literacy in people who declined to participate in the trial for reasons relating to the digital nature of Kidney BEAM. This information will be used to develop strategies to address inequality of access and to improve the reach of Kidney BEAM.

Individual semi-structured interviews will be used to explore participants perspectives in-depth. A topic guide was developed by the qualitative study team (HMLY,JB,RB,EMC, CW, CS), in consultation with PPI representatives, and informed by relevant related literature. Methods of data collection mirrored those of the nested qualitative study within the main trial, except consent was conducted face to face, owing to lack of access to digital technologies. Existing evidence indicates that a range of factors are associated with health inequality and digital exclusion, including increasing age, ethnicity, socio-economic status, employment status, housing status, educational level, geographical location, and disability^10 11^. A bespoke questionnaire was used to collect information on these factors at the start of each interview. Interviews were conducted face to face or via the telephone. Participants were interviewed as close to the declining participation in the trial as possible. Interviews were conducted by JB and HMLY, were scheduled to take up to 60 minutes and were digitally audio-recorded. Participants were offered £20 to re-imburse their time to meet with the qualitative interviewer.

As per the nested interviews, a planned sample size of up to 20 participants was selected to ensure good representation of participants living with different stages of CKD and/or receiving different forms of renal replacement therapy. Maximum variation sampling was initially used to ensure that participants were purposefully diverse^12^, and information from the questionnaire was used to guide sampling. Data collection ceased at the point where information power, guided by an appraisal of the sample specificity, quality of dialogue, and analysis strategy, was achieved^13^. The recorded interviews were professionally transcribed verbatim and subject to analysis according to the framework approach, as described for the nested qualitative interviews for the main trial^14^. These analyses were conducted separately. Information from the bespoke questionnaire was used to explore the data during the charting phase.

**The Polycystic Kidney Disease (PKD) Sub-study**

Recommendations by ‘Kidney Disease: Improving Global Outcomes’ (KDIGO) for individuals affected by and at-risk of PKD include a healthy lifestyle and diet, maintenance of optimal weight, regular exercise, and avoidance of smoking^15^. Nevertheless, despite these recommendations, little is known about the effectiveness of PKD-specific lifestyle modifications in improving HRQoL, physical function and patient activation. Kidney BEAM, which is now widely available for people living with kidney disease in the UK, was an ideal place to create a bespoke education and exercise training module to support people living with PKD to engage with physical activity.

The Kidney BEAM Trial included an exploratory sub-study for 12 adults with PKD. Those patients were directed to complete a PKD-specific education module prior to starting the exercise training as per protocol. Participants completed baseline and 12-week assessments as per protocol and were invited to feedback on the acceptability of the module via semi-structured interviews.

**The Ex-Tab Sub-study**

The recruitment process for the Kidney BEAM Trial excluded numerous participants due to lack of Wi-Fi access, a suitable device to use and reports of participants not feeling confident to access a physical activity focused DHI. This indicated that a significant number of people living with CKD were missing out on the opportunity to participate in the study. There is currently limited digital inclusion research relating specifically to people living with CKD.

The Kidney BEAM Trial includes an exploratory sub-study of *n =* 40 participants from Kings College Hospital who do not have access to a digital device or who are not confident using the technology available to them. The participants were randomised to receive Kidney BEAM as per protocol or the tablet. Participants completed baseline and 12 week assessments as per protocol with the only difference being that a screening tool was included to assess digital health literacy prior to inclusion and consent. All assessments for the participants in the tablet intervention group received a face-to-face assessment, provision of a technology tablet, Wi-Fi access if needed, and training on how to use the device. The tablet intervention group were loaned a technology tablet for 12 (+/- 2 weeks) so that they could access Kidney BEAM and engage in live or on demand classes.

**Haemodialysis Sub-study**

A sub-study was conducted to explore the impact of using Kidney BEAM within an in-centre haemodialysis patient population. This sub-group of patients have a high physical health burden and this will inform the development of Kidney BEAM to further incorporate the needs of different users, including those with multiple long-term conditions and varied health needs who may normally find it more difficult to undertake physical activity.

Evidence in intradialytic exercise programs is difficult to draw robust conclusions from as studies conducted in this area have yielded mixed results historically^16 17^. An easily accessible physical activity DHI with the option for patients to utilise this whilst receiving their dialysis treatment could be beneficial.

A small group of people receiving maintenance haemodialysis (*n*=60) were enrolled to this sub-study, based on the inclusion and exclusion criteria for the main trial with the additional inclusion criteria of:

- Receiving haemodialysis in-centre
- No instability on dialysis in the preceding four weeks prior to baseline visit

i.e., no episodes of intradialytic hypotension, early cessation of sessions for medical reasons or chest pain precipitated by dialysis

In line with the aforementioned goals of the Kidney BEAM trial, the following will be measured pre and post 12-week Kidney BEAM program:

- The Sit-to-Stand 60 (STS60)
- Hand grip strength (in anticipation that some haemodialysis patients will have lower limb amputation or have lower limb disability)
- HRQoL (EQ5D-5L)

Participants will be separated equally into three arms, to evaluate the relevant benefits of supportive interventions in conjunction with Kidney BEAM:

- Arm 1: Access to Kidney BEAM
- Arm 2: Access to Kidney BEAM plus equipment available for use on dialysis (exercise equipment plus IT equipment for access to Kidney BEAM)
- Arm 3: Access to Kidney BEAM, available equipment on dialysis, regular visits from on-site staff – a mixture of both medical and physiotherapy staff – to provide practical tips on use of Kidney BEAM, encouragement and motivation to continue with the program the DHI and supportive exercise options from the physiotherapy team.

This substudy will build the knowledge base relating exercise in people receiving haemodialysis, including ascertaining the level of intervention required to see beneficial changes in this population.

1. ERACODA Working Group. Chronic kidney disease is a key risk factor for severe COVID-19: a call to action by the ERA-EDTA. *Nephrology Dialysis Transplantation* 2021;36(1):87-94.

2. Williamson EJ, Walker AJ, Bhaskaran K, et al. Factors associated with COVID-19-related death using OpenSAFELY. *Nature* 2020;584(7821):430-36.

3. Ribeiro HS, Rodrigues AE, Cantuária J, et al. Post-COVID-19 rehabilitation: a special look at chronic kidney disease patients. *Renal Replacement Therapy* 2021;7(1):1-5.

4. Gabarre P, Dumas G, Dupont T, et al. Acute kidney injury in critically ill patients with COVID-19. *Intensive care medicine* 2020;46:1339-48.

5. Fugazzaro S, Contri A, Esseroukh O, et al. Rehabilitation interventions for post-acute COVID-19 syndrome: a systematic review. *International journal of environmental research and public health* 2022;19(9):5185.

6. Klok FA, Boon GJ, Barco S, et al. The Post-COVID-19 Functional Status scale: a tool to measure functional status over time after COVID-19. *European Respiratory Journal* 2020;56(1)

7. Taylor ML, Thomas EE, Vitangcol K, et al. Digital health experiences reported in chronic disease management: An umbrella review of qualitative studies. *Journal of Telemedicine and Telecare* 2022;28(10):705-17.

8. Stauss M, Floyd L, Becker S, et al. Opportunities in the cloud or pie in the sky? Current status and future perspectives of telemedicine in nephrology. *Clinical Kidney Journal* 2021;14(2):492-506.

9. O’connor S, Hanlon P, O’donnell CA, et al. Understanding factors affecting patient and public engagement and recruitment to digital health interventions: a systematic review of qualitative studies. *BMC medical informatics and decision making* 2016;16:1-15.

10. The Strategy Unit. Improving Digital Health Inclusion: evidence scan 2020 [Available from: <https://www.strategyunitwm.nhs.uk/sites/default/files/2021-04/Digital%20Inclusion%20evidence%20scan.pdf> accessed 29th March 2023.

11. Herrera S, Salazar A, Nazar G. Barriers and Supports in eHealth Implementation among People with Chronic Cardiovascular Ailments: Integrative Review. *International Journal of Environmental Research and Public Health* 2022;19(14):8296.

12. Robinson OC. Sampling in interview-based qualitative research: A theoretical and practical guide. *Qualitative research in psychology* 2014;11(1):25-41.

13. Malterud K, Siersma VD, Guassora AD. Sample size in qualitative interview studies: guided by information power. *Qualitative health research* 2016;26(13):1753-60.

14. Gale NK, Heath G, Cameron E, et al. Using the framework method for the analysis of qualitative data in multi-disciplinary health research. *BMC medical research methodology* 2013;13(1):1-8.

15. KDIGO. Autosomal Dominant Polycystic Kidney Disease (ADPKD) 2023 [Available from: <https://kdigo.org/guidelines/autosomal-dominant-polycystic-kidney-disease-adpkd/> accessed 29th March 2023.

16. Zhang F, Bai Y, Zhao X, et al. Therapeutic effects of exercise interventions for patients with chronic kidney disease: an umbrella review of systematic reviews and meta-analyses. *BMJ open* 2022;12(9):e054887.

17. Greenwood SA, Koufaki P, Macdonald JH, et al. Randomized Trial—PrEscription of intraDialytic exercise to improve quAlity of Life in Patients Receiving Hemodialysis. *Kidney international reports* 2021;6(8):2159-70.
