# Supplementary material for: The effect of a novel, digital physical activity and emotional well-being intervention on health-related quality of life in people with chronic kidney disease: trial design and baseline data from a multicentre prospective, wait-list randomised controlled trial (kidney BEAM)
Source: BMC Nephrol. 2023 May 2;24:122. doi: 10.1186/s12882-023-03173-7 (PMC10152439; doi:10.1186/s12882-023-03173-7)
Supplement: Supplementary file 2 — Supplementary Material 2 [file 12882_2023_3173_MOESM2_ESM.docx]

**Supplementary Material 2: World Health Organisation Trial dataset**

| **Data category** | **Information** |
| --- | --- |
| **Primary registry and trial identifying number** | NCT04872933 |
| **Date of registration in primary registry** | 5^th^ May 2021 |
| **Secondary identifying numbers** | REC number: 21/LO/0243 |
| **Source of funding** | Kidney Research UK |
| **Primary sponsor** | King’s College Hospital NHS Trust |
| **Secondary sponsor(s)** | n/a |
| **Contact for public queries** | sharlene.greenwood@nhs.net |
| **Contact for scientific queries** | sharlene.greenwood@nhs.net |
| **Public title** | The Kidney BEAM Trial |
| **Scientific title** | Multicentre prospective single-blind wait-list randomised controlled trial of the clinical value and cost-effectiveness of an online physical and emotional wellbeing resource for the improvement of health-related quality of life in people with chronic kidney disease: The Kidney BEAM Trial. |
| **Countries of recruitment** | United Kingdom |
| **Health condition(s) or problem(s) studied** | Chronic Kidney Disease (CKD) |
| **Intervention(s)** | - Kidney BEAM - Usual care |
| **Key inclusion and exclusion criteria** | - Patients with established CKD - Aged 18 years+ - Patients who are naïve to Kidney BEAM and who have not participated in a structured exercise programme in the prior 3 months |
| **Study type** | Randomised controlled trial |
| **Date of first enrolment** | 01/06/2021 |
| **Target sample size** | 304 participants |
| **Recruitment status** | Recruitment completed |
| **Primary outcome(s)** | Mental Composite Score (MCS) of KDQoL-36 quality of life questionnaire at 12 weeks. |
| **Key secondary outcomes** | - EQ5D-5L questionnaire - Patient Activation Measure (PAM-13) - The Patient-Health Questionnaire-4 - Chalder Fatigue Questionnaire -- physical and mental fatigue - Work and Social Adjustment Scale (WSAS) - Functional capacity (Sit-to-Stand 60) - Global Physical Activity Questionnaire (GPAQ) - Quality of life (KDQOL-36 physical composite score, energy/fatigue, burden of kidney disease, role physical, physical functioning, mental health, bodily pain, role emotional, social functioning, general health) - Patient engagement with Kidney BEAM - Healthcare utilisation - Adverse events |
