# Supplementary material for: The effect of a novel, digital physical activity and emotional well-being intervention on health-related quality of life in people with chronic kidney disease: trial design and baseline data from a multicentre prospective, wait-list randomised controlled trial (kidney BEAM)
Source: BMC Nephrol. 2023 May 2;24:122. doi: 10.1186/s12882-023-03173-7 (PMC10152439; doi:10.1186/s12882-023-03173-7)
Supplement: Supplementary file 3 — Supplementary Material 3 [file 12882_2023_3173_MOESM3_ESM.docx]

**Supplementary Material 3: Full inclusion and exclusion criteria.**

| **INCLUSION CRITERIA** | **EXCLUSION CRITERIA** |
| --- | --- |
| Individuals with established CKD | Weight < 50kg |
| Adults aged 18yrs+ | Insufficient understanding of the trial |
| Access to a Wi-Fi-enabled device | Self-reported participation in a structured exercise programme or Kidney BEAM within previous 3 months |
| Able to understand written English language | Active infection |
| Written or virtual informed consent | Uncontrolled arrhythmias |
|  | Unstable angina or heart attack within the 3 months |
|  | Persistent uncontrolled hypertension (systolic blood pressure >180 mm Hg or diastolic blood pressure >110 mm Hg) |
|  | Recent (within the last 3 months) stroke or transient ischaemic attack |
|  | Receiving palliative care for advanced terminal cancer |
|  | Patients with peripheral vascular or musculoskeletal disease, who the investigator deems unable to carry out a physical activity intervention. |
|  | Any other health condition considered by the local Principal Investigator in which exercise therapy will be contraindicated |
