# Supplementary material for: The effect of a novel, digital physical activity and emotional well-being intervention on health-related quality of life in people with chronic kidney disease: trial design and baseline data from a multicentre prospective, wait-list randomised controlled trial (kidney BEAM)
Source: BMC Nephrol. 2023 May 2;24:122. doi: 10.1186/s12882-023-03173-7 (PMC10152439; doi:10.1186/s12882-023-03173-7)
Supplement: Supplementary file 4 — Supplementary Material 4 [file 12882_2023_3173_MOESM4_ESM.pdf]

beam King's College Hospital NHS
NHS Foundation Trust

Patient Informed Consent Form (Main Study)

A study to evaluate the clinical value and cost of an online physical and emotional wellbeing resource for the improvement of health-related quality of life in people with chronic kidney disease: The Kidney Beam Trial.

IRAS Number: IRAS 291403

Chief Investigator: Dr Sharlene Greenwood

Patient Name:

Please initial box

**1**	I confirm that I have read the information sheet dated 20.10.2021 (version 3.0) for the above study. I have had the opportunity to consider the information, ask questions and have had these answered satisfactorily.	☐
**2**	I understand that my participation is voluntary and that I am free to withdraw at any time without giving any reason, without my medical care or legal rights being affected.	☐
**3**	I understand that the patient interviews will be recorded and that these are optional. I may, or may not, be invited to take part in these.	☐
**4**	I understand that relevant sections of my medical notes, contact details and data collected during the study, will be shared with individuals from the central research site, which is also the sponsor of the trial (King's College Hospital NHS Trust). This will also include responsible persons authorized by the sponsor, from regulatory authorities or from an NHS Trust, where it is relevant to my taking part in this research. I give permission for these individuals to have access to my records.	☐
**5**	I consent to the information collected about me, including audio recordings (where participants have been invited to interview), to be used to support other research in the future, and that this may be shared anonymously with other researchers.	Yes ☐ No ☐
**6**	I agree to my General Practitioner being informed of my participation in the study.	Yes ☐ No ☐
**7**	I agree to be contacted about ethically approved future research.	Yes ☐ No ☐

8 I agree to take part in the above study. Yes ☐ No ☐

9 I wish to receive the final report at the end of the study. Yes ☐ No ☐

Not Applicable for Kings College Hospital Participants

10 I understand that my contact details will be forwarded to the Kings College Hospital research team who will contact me to schedule and conduct assessment study visits. ☐

11 Email address:
(For follow up communication on completion of this consent form)

12 Which hospital do you attend?

Name of Participant	Date	Signature
Name of Person taking consent	Date	Signature

When completed: 1 for participant; 1 for researcher site file; 1 (original) to be kept in medical notes.

Participant Information Sheet
Main Study

A study to evaluate the clinical value and cost of an online physical and emotional wellbeing resource for the improvement of health-related quality of life in people with chronic kidney disease: The Kidney Beam Trial.

PART 1

We'd like to invite you to take part in our research study.
Joining the study is entirely up to you, before you decide we would like you to understand why the research is being done and what it would involve for you.

One of our team will go through this information sheet with you, to help you decide whether or not you would like to take part and answer any questions you may have. Please feel free to talk to others about the study if you wish.

The first part of the Participant Information Sheet tells you the purpose of the study and what will happen to you if you take part.

The second part will give you more detailed information about the conduct of the study. Please ask if anything is unclear.

What is the purpose of the study?

This study will examine the clinical value and cost-effectiveness of an online physical and emotional wellbeing resource for the improvement of health-related quality of life in people with CKD.

Kidney BEAM is a new online service to support patients with kidney disease to stay physically active. This is led by specialist kidney professionals. We will examine whether Kidney BEAM can improve physical and mental well-being through questionnaire assessment and some physical tests. We will also explore the patient’s perception to Kidney BEAM and their experiences of using the platform.

Why have I been invited?

You have been invited to take part in the study because you have a medical condition called Chronic Kidney Disease. This means that either your kidneys work less well than expected or that your kidneys leak protein or blood into your urine. We expect approximately 304 patients will take part in this study that is open to all patients with Chronic Kidney Disease.

Do I have to take part?

It is up to you to decide. We will describe the study and go through this information sheet, which we will then give to you. You will be able to keep this information sheet and think about taking part. You

are free to discuss the information with anyone, including your family and friends. If you agree, we will then ask you to sign a consent form to show you have agreed to take part. You are free to withdraw at any time, without giving a reason. This would not affect the standard of care you receive.

What will happen to me if I take part?

You will see one of the physiotherapists or research nurses who will answer any questions that you may have. If you agree to take part, we will ask you to sign a consent form and you will give you a copy to keep. We register your name on the computer to determine which group you will be in, either waiting list or non-waiting list. We will allocate a unique study identifier for you to ensure anonymity and confidentiality of your data. If you are assigned to the non-waitlist group, you will be asked to attend a total of 3 virtual assessments whilst in the study: at baseline, at 12 weeks then at 6 months. You will be offered the online intervention straight after completing your baseline visit. If you are assigned to the waitlist group, you will be asked to still do your assessments at baseline and 12 weeks but will only start the online intervention after the 12-week assessment. During assessment, we will ask your referring site to provide us with your contact details, up-to-date blood test results and vital signs like heart rate and blood pressure (from your medical records). We will contact you from the main site and ask you to complete 6 questionnaires, which will likely take 40-50 mins in total to complete, and can be done with breaks in between, and ask you to perform a 60 second sit-to-stand test (this is a test that measures how many times you can perform a sit to stand movement in 60 seconds). You will be asked about your physical activity to assess whether you are meeting current physical guidelines. We will also ask for your permission to look at your medical records to see your medical history and recent blood test and vital signs results.

After completing the intervention, we will ask some participants to have an interview with a physiotherapist to talk about your experience of the study. This will not take longer than 1 hour and it is up to you whether you choose to take part in this part of the study when invited. You will not be invited to attend more than one interview during the study. The interviews will explore experiences and the impact of the intervention. The interviews will be recorded. The audio recordings will be transcribed into written form, and entered into a password-protected database, de-identified and destroyed after completion of the study. The audio recordings will be deleted following transcription. Any publications of direct quotes from the interview will not be identifiable.

If you have had a COVID-19 hospital admission prior to agreeing to take part in the study, we will ask you to complete an additional short questionnaire about your hospital admission and recovery.

Please see the table below that shows what is involved at each study visit.

PROCEDURE	Screen Visit	Baseline Visit	Week 12 Visit	6-month follow-up (non-waitlist group only)
Patient information and informed consent	X			
Demographic data blood test results and medical history from medical records	X			
Fatigue Severity Score (Chalder Fatigue questionnaire)		X	X	
Functional impairment (WSAS Questionnaire)		X	X	
Quality of life (Kidney Disease Quality of Life-36 and European Quality of life 5 Dimension-5 Level questionnaires)		X	X	X

Functional capacity (Sit To Stand 60)		X	X	
Global Physical Activity Questionnaire (GPAQ), **Physical Health Questionnaire (PHQ9) and additional questions on meeting current physical activity guidelines.**		X	X	
Patient Activation Measure			X	
Patient Interview (by invitation)			X	
Kidney Beam platform metrics (Physical activity mins and engagement)			X	X
Healthcare utilisation questionnaire		X	X	X
Post-COVID functional assessment tool questionnaire (for patients with history of COVID-19 hospital admission)		X	X	X

What will the Kidney BEAM intervention involve?
Kidney BEAM is an online platform where you will have access to live and on-demand movement or physical activity classes led by renal physiotherapists, renal counsellors and qualified yoga instructors. It offers a new way to improve your physical activity in the comfort of your own home. As part of this study, we will invite you to attend 2 structured exercise and education classes per week (24 sessions in total) over the 12-week period. These classes will be offered live or can be done on-demand at a time that suits you. The classes will include a variety of exercises to improve fitness, strength and balance. You will also be able to choose other classes or activity you want to attend, either on the online platform or off the platform, and will be encouraged to accumulate 150 mins/week of moderate intensity aerobic activity or 75 mins/week of vigorous activity. A physiotherapist will guide you with this activity, and there is an online dashboard to record all types of activity.

What are the possible benefits of taking part?
You will have access to the free online resource to help improve physical activity and mental health. Your participation will also provide data to support clinical commissioning of the Kidney BEAM digital health intervention platform for management of patients with kidney disease in the future.

We cannot promise the study will help you but the information we get from this study will help improve the future treatment of people with chronic Kidney Disease.

What are the possible disadvantages and risks of taking part?
Potential risk of injury may happen during any type of physical activity. You will be guided by qualified instructors to prevent this. An assessment will also be done prior to you starting to decide a safe exercise for you to do**.**

Who is organising and funding this study?

The person in charge of this study is: Dr Sharlene Greenwood. The study is funded by Kidney Research UK and is being sponsored by Kings College Hospital NHS Foundation Trust.
The sponsors of this study will pay your hospital for including you in this study.

How have patients and the public been involved in this study?
In designing this study, we have taken into account patient opinions on the frequency of participant visits and the tests that we will carry out.

Who has reviewed this study?
All research in the NHS is looked at by an independent group of people, called a Research Ethics Committee, to protect your interests. This study has been reviewed and given favourable opinion by _______________Research Ethics Committee. It has also been approved by the Health Research Authority and each local hospital will also give confirmation that the study can go ahead.

Expenses and Payments
There are no funds available for payments to those participating in this study.

This completes Part 1 of the Information Sheet.
If the Information in Part 1 has interested you and you are considering participation, please continue to read the additional information in Part 2 before making any decision.

PART 2

What if new information becomes available?
Sometimes we get new information about the treatment being studied. If this happens, your study doctor will tell you and discuss whether you should continue in the study. If you decide not to carry on, your study doctor will make arrangements for your care to continue. If you decide to continue in the study he/she may ask you to sign an agreement outlining the discussion.

This new information that becomes available might specifically affect you and your health. If this happens, your study doctor might consider that you should withdraw from the study. He/she will explain the reasons for withdrawing from the study and arrange for your care to continue.
If the study is stopped for any other reason, we will tell you and arrange for your continuing care.

What will happen if I don't want to carry on with the study?
If you do not wish to continue taking part, you are free to withdraw at any time. If possible, we will ask your reason for withdrawal only for purpose of documentation. No further study data will be collected after formally withdrawing your consent.

What if there is a problem?
If you have a concern about any aspect of this study, you should ask to speak to your study doctor who will do their best to answer your questions (contact details can be found at the end of this sheet). If you remain unhappy and wish to complain formally, you can do this through the NHS Complaints procedure by contacting your local Patient Advice Liaison Service (PALS) office. Details of your local office can be obtained by asking your study doctor, GP, telephoning your local hospital or looking on the NHS choices website. http://www.nhs.uk/pages/home.aspx

Every care will be taken in the course of this study. However, in the unlikely event that you are injured by taking part, compensation may be available.

In the event that something does go wrong and you are harmed during the research and this is due to someone's negligence then you may have grounds for a legal action for compensation against Kings College Hospital NHS Trust but you may have to pay your legal costs.

Regardless of this, if you wish to complain, or have any concerns about any aspect of the way you have been approached or treated by members of staff or about any side effects (adverse events) you may have experienced due to your participation in the study the normal National Health Service complaints mechanisms are available to you. Please ask your study doctor if you would like more information on this.

Will my taking part be kept confidential?
All information collected about you during the study will be kept strictly confidential and will be stored securely and handled according to data protection guidelines.

The research team will seek information from your doctors and from NHS and other central registries about any serious illnesses that may occur. This requires your name, date of birth and NHS number. All information received will be used, in confidence, only for medical research purposes and for routine regulatory and audit purposes.

Authorised individuals from regulatory authorities, the sponsor's representatives, King's Clinical Trials Unit and NHS bodies may look at the study information to ensure that the study is being carried out correctly but they will be bound by rules of confidentiality.

The organisations listed above will keep information about you confidential and secure. Your name will not be used in any reports about the study and all data is stored in accordance with the principle of the Data Protection Act DPA 2018.

Involvement of the General Practitioner/Family Doctor (GP)
With your consent, your GP will be informed of your involvement in the trial. Any other medical practitioners who treat you, e.g. should you be admitted to hospital for any reason, will also be informed.

What will happen to the results of the research study?

It is intended that the results of the study will be reported and disseminated at conferences and in peer-reviewed scientific journals. All proposed publications will be discussed with and reviewed by the Sponsor prior to publishing other than those presented at scientific forums/meetings. You will not be identifiable from any report or publication placed in the public domain.

We will send you a summative report of the study by email or post.

How we will use your data

We will need to use information from your medical records for this research project.

This information will include your:

- Name
- NHS number
- Contact details
- Medical records

People will use this information to do the research or to check your records to make sure that the research is being done properly.

People who do not need to know who you are will not be able to see your name or contact details. Your data will have a code number instead.

We will keep all information about you safe and secure.

Once we have finished the study, we will keep some of the data so we can check the results. We will write our reports in a way that no-one can work out that you took part in the study.

What are your choices about how your information is used?

You can stop being part of the study at any time, without giving a reason, but we will keep information about you that we already have.

- We need to manage your records in specific ways for the research to be reliable. This means that we won't be able to let you see or change the data we hold about you.

You can find out more about how we use your information:

- on the Health Research Authority website www.hra.nhs.uk/information-about-patients/
- in a leaflet called: HowWeWillUseYourData KCH V1 (21-11-19) – available from the study team
- at our website https://www.kch.nhs.uk/about/corporate/data-protection
- by emailing our Data Protection Officer on kch-tr.dpo@nhs.net

We will ask you if you are happy, and consent, to be contacted about future research.

Thank you

Thank you for considering taking part and taking the time to read this information sheet.

If you decide to take part in the study, we will give you a copy of the information sheet and a signed consent form to keep.

Further information and contact details

INVOLVE, Alpha House, University of Southampton Science Park, Chilworth, Southampton, SO16 7NS
Telephone: 023 8059 5628 Email: involve@nihr.ac.uk

Local Contacts:
Your doctor .... Tel: ....
Your nurse/study coordinator... Tel: ....

Or you can direct questions to the lead Investigator for the trial:

Dr Sharlene Greenwood
Renal Unit, King’s College Hospital, London, SE5 9RS
Telephone: 0203 299 6233
Email: sharlene.greenwood@nhs.net
