# Supplementary material for: The effect of a novel, digital physical activity and emotional well-being intervention on health-related quality of life in people with chronic kidney disease: trial design and baseline data from a multicentre prospective, wait-list randomised controlled trial (kidney BEAM)
Source: BMC Nephrol. 2023 May 2;24:122. doi: 10.1186/s12882-023-03173-7 (PMC10152439; doi:10.1186/s12882-023-03173-7)
Supplement: Supplementary file 5 — Supplementary Material 5 [file 12882_2023_3173_MOESM5_ESM.docx]

**Supplementary Material 5: Kidney BEAM Education Sessions.**

| Week | Topic for education |
| --- | --- |
| 1 | The Role of Exercise in Chronic Kidney Disease |
| 2 | Personalising Exercise Plans |
| 3 | Goal Setting |
| 4 | Staying Active for the Long Term |
| 5 | Barriers and How to Overcome Them |
| 6 | The Role of Your Kidneys |
| 7 | Breathlessness and Fluid Intake |
| 8 | Fatigue and Anaemia |
| 9 | The Impact of High Blood Pressure |
| 10 | Diabetes and Kidneys |
| 11 | Nutrition and Diet for your Kidneys |
| 12 | Renal Counselling |
