# Supplementary material for: The effect of a novel, digital physical activity and emotional well-being intervention on health-related quality of life in people with chronic kidney disease: trial design and baseline data from a multicentre prospective, wait-list randomised controlled trial (kidney BEAM)
Source: BMC Nephrol. 2023 May 2;24:122. doi: 10.1186/s12882-023-03173-7 (PMC10152439; doi:10.1186/s12882-023-03173-7)
Supplement: Supplementary file 6 — Supplementary Material 6 [file 12882_2023_3173_MOESM6_ESM.docx]

**Supplementary Material 6 – Topic guide for the main interviews.**

1. What first made you interested in Kidney BEAM?
2. Tell me what you think about Kidney BEAM?
3. Can you tell me about your exercise/ physical activity before beam?
4. Can you tell me how you have been using Kidney BEAM?
5. Did you come up against any barriers or challenges using Kidney BEAM?
6. How did you overcome these concerns/ barriers?
7. What support was available to help you be more physically active
8. Do you think any additional support might have helped you ?
9. Did you do any physical activity or exercise outside of Kidney BEAM (offline)?
10. Did your participation in the study influence your daily life
11. What parts of Kidney BEAM helped you stay involved from the beginning to the end of the programme?
12. What changes could have helped you stay more involved for the full 12 weeks?
13. What improvements were you most hoping to see as a result of taking part in Kidney BEAM?
14. Some people find living with CKD can impact their mental wellbeing. Can you tell us a little bit about the impact living with CKD on your daily life?
15. Has using Kidney BEAM influenced your mental wellbeing in any way?
16. Do you plan to continue to use Kidney BEAM when the study is over?
17. What challenges or facilitators, if any, do you think you may face when continuing to use Kidney BEAM in the longer term?
18. Would you recommend Kidney BEAM to other people with kidney disease? What would you tell them about it?
